# Analysis of the aspartic protease gene family in *Nicotiana benthamiana* and its application in recombinant protein expression

**DOI:** 10.3389/fpls.2026.1778448

**Published:** 2026-03-16

**Authors:** Yu’e Li, Tao Liu, Lianghua Zhu, Rongrong Wu, Youjie Liu, Huijuan Zhou, Wei Guo, Shiqiang Zhang, Sayed Abdul Akher, Hongwei Piao, Jianqi Zhang, Kexin Liang, Zhenqi Zhao, Yongfeng Guo, Zhe Jin, Zenglin Zhang

**Affiliations:** 1 Technology Research and Development Center of Jilin Tobacco Industry Co., Ltd, Changchun, China; 2 Tobacco Research Institute, Chinese Academy of Agricultural Sciences, Qingdao, China; 3 Qingdao Municipal Key Laboratory of Plant Molecular Pharming, Tobacco Research Institute, Chinese Academy of Agricultural Sciences, Qingdao, China

**Keywords:** aspartic protease, bioreactor, gene family, *Nicotiana benthamiana*, recombinant protein platform

## Abstract

**Introduction:**

Aspartic proteases (APs) play crucial roles in plant growth, stress responses, and protein metabolism. However, endogenous APs in *Nicotiana benthamiana*, a key plant molecular farming platform, pose a significant challenge by degrading valuable recombinant proteins, thereby limiting production yields.

**Methods:**

A comprehensive understanding of the AP gene family in *N. benthamiana* is essential for developing strategies to mitigate this degradation. In this study, multiple bioinformatics approaches were employed to identify the NbAPs gene family in the *Nicotiana benthamiana* genome. The analysis encompassed the protein characteristics, phylogenetic relationships, gene structures, conserved motifs, gene duplication events, and chromosomal distribution of family members. Additionally, the role of NbAPs members in recombinant protein expression was investigated.

**Results:**

We performed a genome-wide analysis and identified 89 Aspartic Protease (NbAP) genes in *N. benthamiana*. Phylogenetic analysis classified these genes into three subfamilies-typical, nucellin-like, and atypical-revealing both evolutionary conservation and diversification. The NbAP members exhibited diverse gene structures, conserved motifs, and subcellular localizations, with significant enrichment predicted for the vacuole and chloroplasts. Segmental duplication was identified as the primary mechanism of NbAP family expansion. Promoter analysis revealed the presence of cis-elements associated with stress responses, hormone regulation, and developmental processes. Transcriptome and RT-qPCR analyses showed dynamic expression patterns of NbAPs following *Agrobacterium* infiltration, with genes such as *NbAP46*, *NbAP47*, or *NbAP79* showing sustained upregulation. Functional validation using Virus-Induced Gene Silencing (VIGS) demonstrated that silencing *NbAP46*, *NbAP47*, or *NbAP79* significantly enhanced the accumulation of transiently expressed GFP protein.

**Discussion:**

This study provides the first comprehensive genomic identification and characterization of the AP family in *N. benthamiana*. We highlight the diversity of NbAPs and their potential biological roles, and experimentally validate that specific members negatively influence recombinant protein stability. Silencing these proteases leads to enhanced accumulation of foreign proteins. Overall, our findings establish a foundational resource and identify potential targets for genetic engineering to further optimize *N. benthamiana* as a superior bioreactor for plant molecular farming.

## 1 Introduction

APs, a widespread class of hydrolases in eukaryotes, derive their name from the two conserved aspartic acid residues at their active sites (Norero et al., 2022). These enzymes play central roles in plant growth and development, stress responses, and protein metabolism (Wang et al., 2024). According to the MEROPS database (Rawlings et al., 2018), plant APs primarily belong to the A1 family (pepsin-like family) and can be further divided into three categories: typical, atypical, and nucellin-like (Faro and Gal, 2005). The structure of typical plant APs is the most distinctive: their pro-proteins contain a N-terminal hydrophobic signal peptide (guiding endoplasmic reticulum localization), an inhibitory pro-segment (which maintains zymogen stability and participates in intracellular targeting), two mature protease domains (each contributing one catalytic Asp residue), and a plant-specific insert (PSI) domain (Norero et al., 2022). The PSI, structurally classified as a saposin-like protein (SAPLIP) domain, is involved not only in proper protein folding (Muñoz et al., 2010) but also in diverse biological processes (such as pathogen defense, salt and drought stress responses) and subcellular localization (Bi et al., 2005; Phan et al., 2011; Sebastián et al., 2020). Crucially, the PSI has been identified as one of the key signals regulating the vacuolar sorting of typical Aps, and its glycosylation status can directly affect the trafficking route of the pro-protein after passage through the Golgi apparatus (Vitale et al., 2019).

In plants, the expression of AP exhibits significant spatiotemporal and organ specificity. For example, the floral organs of Asteraceae plants, such as *Cynara cardunculus* and *Silybum marianum*, are rich in highly active APs that participate in stylar transmitting tissue development, pollination, and petal senescence (Vairo Cavalli et al., 2013). Owing to their potent casein-hydrolyzing activity, these “floral APs” (e.g., cardosins A/B and cyprosins) have been extensively studied as plant-derived alternatives to cheese rennet (Pimentel et al., 2007). Interestingly, different AP isoforms within the same species may localize to distinct subcellular compartments. For example, in *Cynara cardunculus*, cardosin B is secreted to the apoplast, while cardosin A is stored in the storage vacuoles of stigma papilla cells. However, when transiently expressed in heterologous systems such as *N. benthamiana* leaves, both proteins predominantly localize to the lytic vacuole (Pereira et al., 2008; Almeida et al., 2012). This localization plasticity suggests that plant APs utilize multiple vacuolar sorting mechanism, guided by targeting signals such as the C-terminal vacuolar sorting determinant (ctVSD) and the PSI domain, which exhibit a functional hierarchy where the ctVSD is the primary signal for vacuolar sorting, and the PSI acts as an auxiliary regulatory element, especially compensating when ctVSD integrity is compromised (Colombo et al., 2025).

Recent research on APs from *Silybum marianum* flowers (silipepsin 1/2, AP-Sm1/AP-Sm2) provides direct evidence for the regulatory role of the PSI domain (Colombo et al., 2025). Although the AP-Sm1 and AP-Sm2 pro-proteins share 79% sequence similarity and both contain the conserved ctVSD with the sequence “VGFAEAA”, when their mRFP fusion proteins were transiently expressed in *N. benthamiana* leaves, AP-Sm2 still localized to the vacuole even when the ctVSD function was disrupted, whereas AP-Sm1 was mis-sorted to the apoplast. This difference is attributed to distinct glycosylation patterns within their PSI domains: the PSI of AP-Sm1 contains an N-glycosylation site (Asn397), while AP-Sm2 lacks this modification. Studies indicate that PSI glycosylation might determine the protein’s trafficking fate by altering its interaction with sorting receptors (e.g., vacuolar sorting receptors, VSRs) or by affecting endoplasmic reticulum export efficiency (Cao et al., 2019; Colombo et al., 2025). This discovery not only deepens the understanding of plant protein sorting mechanisms but also provides molecular targets for optimizing the *N. benthamiana* recombinant protein expression system.

*Nicotiana benthamiana* is widely used as a “plant factory” platform for recombinant protein production; however, a major bottleneck is the degradation of recombinant products by endogenous APs, which limits overall yield (Tschofen et al., 2016; Yang et al., 2025). Following agroinfiltration of *N. benthamiana* leaves, foreign genes can efficiently express high-value proteins such as antibodies, vaccines, and enzymes (Buyel, 2023; Vo and Trinh, 2025). However, the plant’s inherent protease system (especially Aps) can degrade these recombinant proteins, leading to reduced yield or loss of activity. The degradation risk posed by APs is likely closely associated with their subcellular localization. Vacuole-localized APs are normally segregated from recombinant proteins in the cytosol or secretory pathway by the tonoplast. However, when cells experience osmotic stress or undergo programmed cell death, vacuole rupture can lead to enzyme leakage and widespread degradation. In contrast, APs localized to apoplast can degrade recombinant proteins during transport or after secretion, posing the most immediate threat. Therefore, comprehensive characterization of the AP gene family in *N. benthamiana* is crucial for developing strategies to minimize recombinant protein degradation and enhance production yields.

A genome-wide identification of the AP family members in *N. benthamiana*, clarifying their homology with known APs through phylogenetic analysis, and prediction of the PSI structural features for each member, will systematically elucidate the degradative potential within the *N. benthamiana* AP family. This will facilitate the development of targeted inhibition strategies, thereby promoting quality enhancement and efficiency improvement in the plant-based biopharmaceutical industry.

## 2 Materials and methods

### 2.1 Plant materials and growth conditions

The experimental materials used in this study were *N. benthamiana* seeds preserved by the Chinese Academy of Agricultural Sciences. Plants were grown under a 16-h/8-h (light/dark) photoperiod at 25 °C. Four-week-old plants were subjected to *Agrobacterium* infiltration.

### 2.2 Identification and sequence analysis of the NbAP gene family

To identify members of the APs gene family, we first downloaded the protein sequences of all Arabidopsis APs family members from the TAIR database (www.arabidopsis.org). Analysis of these APs family members using the Pfam database v38.1 (http://pfam.xfam.org/) indicated that they generally possess the conserved domain PF00026. We then downloaded the *N. benthamiana* genome and protein files (NbeHZ1 version) from the Nicomics website (http://lifenglab.hzau.edu.cn/Nicomics/index.php). The hidden Markov model (HMM) file for the APs gene family was retrieved and downloaded from the Pfam database (http://pfam.xfam.org/) using the hmmsearch program with an E-value threshold of 1e-20. The protein sequences of the AtAP family were used as queries to search for potential NbAP candidates through BLAST with an E-value of 1e-5. Protein sequences obtained from both methods were combined, deduplicated and subsequently uploaded to CDD (https://www.ncbi.nlm.nih.gov/Structure/bwrpsb/bwrpsb.cgi) and SMART (http://smart.embl.de/smart/set_mode.cgi?NORMAL=1) for domain validation to remove sequences lacking the PF00026 domain (Finn et al., 2014). In cases where multiple splice variants existed, only the longest transcript isoform was retained for further analysis. Various physicochemical properties of the NbAP protein members, including protein length, molecular weight, isoelectric point, aliphatic amino acid index, and grand average of hydropathicity (GRAVY), were determined using the ProtParam tool available on the Expasy online platform (http://www.expasy.org/tools/protparam.html) (Gasteiger et al., 2003). The *NbAP* genes were named based on their chromosomal location.

### 2.3 The evolutionary tree analysis of the NbAP gene family members

The phylogenetic tree of AP proteins was constructed using MEGA11. Sequence alignment was performed using the ClustalW method, excluding non-conserved regions outside the aligned domains. The phylogenetic tree was constructed using the maximum likelihood method with a bootstrap value of 1000 (Kumar et al., 2018).

### 2.4 Analysis of gene structure, protein domains, and conserved motifs

The online tool MEME (http://meme-suite.org/) was used to predict conserved motifs in NbAP proteins. The motif length was set to 6–100 amino acids, with a maximum of 10 motifs to identify, while maintaining default values for other parameters (Bailey et al., 2015). Batch CD-Search (https://www.ncbi.nlm.nih.gov/Structure/bwrpsb/bwrpsb.cgi) was used for the NbAP family, and their protein-conserved domains were visualized using TBtools. Gene structure analysis was performed based on the genomic DNA and CDS sequences of the NbAPs gene family members, and visualization was conducted using the online gene structure display server 2.0 (https://gsds.gao-lab.org/Gsds_help.php).

### 2.5 Identification of cis-acting regulatory elements in the NbAP genes

The 2000 bp region upstream of the transcription start site was extracted from the genome sequence file. The cis-regulatory elements (CAREs) in these promoter regions were analyzed using PlantCARE software (http://bioinformatics.psb.ugent.be/webtools/plantcare/html/) (Rombauts et al., 1999; Lescot et al., 2002). Finally, all identified elements in the promoter regions of the *NbAP* genes were presented as a heatmap using TBtools.

### 2.6 Chromosomal localization and collinearity analysis

Information on the start and stop positions of AP genes was extracted from the *N. benthamiana* NbeHZ1 genome GFF3 file using TBtools and subsequently mapped onto the corresponding chromosomes. For intra-species analysis of NbAPs, the Fasta Tools and Blast tools in TBtools were used, incorporating chromosome length files, gene location files, and corresponding alignment files (Chen et al., 2020). APs were analyzed using the One Step MCScanX tool with an E-value set to 1e-5. The resulting collinearity file was used for segmental duplication gene analysis, and the tandem file was used to detect tandemly repeated gene analysis (Wang et al., 2012). The non-synonymous to synonymous substitution ratios (Ka/Ks) of homologous gene pairs within the NbAP gene family was calculated using KaKs_Calculator 3.0 software (Zhang, 2022).

### 2.7 VIGS technology validation of AP members involved in recombinant protein stability experiments

Based on conserved sequence features, we designed VIGS fragments for *NbAP46, NbAP47, and NbAP79* (**Supplementary Table 2**) and constructed them into the VIGS vector pTRV2 using homologous recombination technology. These were then co-transformed with pTRV1 into *N. benthamiana*. Briefly, Single colonies of Agrobacterium tumefaciens harboring the pTRV1 or recombinant pTRV2 plasmids were separately inoculated into YEP liquid medium supplemented with appropriate antibiotics and cultured at 28 °C with shaking until the mid-logarithmic growth phase. Bacterial cells were collected by centrifugation and resuspended in infiltration buffer containing 10 mM MES and 100 μM acetosyringone. The two bacterial suspensions were mixed at a 1:1 volume ratio, and the final OD_600_ was adjusted to 0.5–0.8. After incubating the mixed suspension at room temperature in the dark for 2–3 hours, it was injected into the abaxial side of leaves of approximately 4-week-old *N. benthamiana* seedlings using a syringe. After 20 days, RT-qPCR was performed on newly emerged young leaves to analyze the transcript levels of the target genes. Simultaneously, leaves with silenced target genes were used as the chassis for transient expression of GFP protein, which was subsequently detected.

### 2.8 RT-qPCR experiment

Total RNA was extracted from *N. benthamiana*, treated with DNase I, and reverse transcribed into cDNA using PrimeScript RT Master Mix. Using this cDNA as a template, RT-qPCR was performed with target gene primers (**Supplementary Table 1**). The *NbActin* gene(JQ256516.1) was used as the internal reference, and data were calculated and analyzed using the 2^−ΔΔCt^ method (Schmittgen and Livak, 2008). Three replicates were set up for each sample.

### 2.9 Transient expression of GFP in *Nicotiana benthamiana*

The pTRBO::GFP plasmid was introduced into *Agrobacterium tumefaciens* strain GV3101. Positive transformants were selected on YEP solid medium containing appropriate antibiotics at 28 °C for 48 hours. Bacterial cells were harvested and resuspended in infiltration buffer to a final OD_600_ of 0.5. The bacterial suspension was kept at room temperature in the dark for 3 hours before being infiltrated into the abaxial side of leaves from 4-week-old *N. benthamiana* plants using a sterile syringe. After inoculation, plants were maintained under 25 °C with a 16/8-hour light/dark photoperiod. GFP fluorescence (wavelength 380nm ultraviolet light intensity at 38 mm is 8000 μW/cm²) was observed and leaf samples were collected at specified time points for subsequent analysis.

## 3 Results

### 3.1 Identification and basic characterization analysis of the N. benthamiana APs family

A total of 129 candidate AP sequences were identified. Subsequently, 89 genes were identified to contain a complete ASP domain (PF00026) by NCBI-CDD (v3.20) and SMART. (**Table 1**). Analysis of their physicochemical properties using protein analysis tools revealed that the protein lengths ranged from 232 to 666 amino acids (shortest: *NbAP14*/232 aa; longest: *NbAP75*/666 aa). Their molecular weights ranged from 26,049.71 to 74,023.16 Da (smallest: *NbAP14*/26.05 kDa; largest: *NbAP75*/74.02 kDa). The theoretical isoelectric point (pI) spanned from 4.57 to 9.52 (most acidic: *NbAP33*/pI 4.57; most basic: *NbAP49*/pI 9.52). The instability index values ranged from 22.38 to 51.90 (most stable protein: *NbAP17*/22.38; most unstable: *NbAP44*/51.90). The aliphatic index ranged from 70.89 to 97.65 (lowest: *NbAP47*/70.89; highest: *NbAP65*/97.65). The grand average of hydropathicity (GRAVY) ranged from -0.330 to 0.268 (most hydrophilic: *NbAP47*/-0.324; most hydrophobic: NbAP62/0.256). Furthermore, subcellular localization predictions indicated the main compartments as: Vacuole (25 members, e.g., *NbAP1, NbAP15, NbAP20*) and Chloroplast (31 members, e.g., *NbAP2, NbAP4, NbAP7*). Extracellular localization included *NbAP32, NbAP53, NbAP56, NbAP73*. Endoplasmic reticulum localization included *NbAP30, NbAP38*. Plasma membrane localization included *NbAP3, NbAP6, NbAP10*, etc. (14 members). Nucleus localization included *NbAP35, NbAP45, NbAP58*, etc. (7 members). Vacuole-localized members (28%) may participate in recombinant protein degradation (e.g., *NbAP16, NbAP23, NbAP54*); extracellularly localized enzymes (e.g., *NbAP32, NbAP53*) pose the highest risk for degrading recombinant products in the secretory pathway. The enrichment of *N. benthamiana* AP family members in the vacuole (28%) and chloroplasts (35%) highlights their important role in plant protein degradation and stress responses.

**Table 1:** Detailed information of the 89 Predicted NbAPs proteins in *Nicotiana benthamiana*.

Gene locus	Gene symbol	Gene position	Protein length(aa)	Molecular weight(Da)	Theoretical PI	Instability index	Aliphatic index	Grand average of hydropathicity	Subcellular localization
Nbe01g09880.1	NbAP1	Nb01	492	52636.57	8.86	37.79	78.84	-0.029	Vacuole
Nbe01g10430.1	NbAP2	Nb01	438	46793.29	9.36	45.45	79.68	-0.013	Chloroplast
Nbe01g12550.1	NbAP3	Nb01	653	71861.36	7.6	47.97	85.68	-0.12	Plasma Membrane
Nbe01g15250.1	NbAP4	Nb01	506	55126.01	5.83	31.36	89.13	0.018	Chloroplast
Nbe01g21350.1	NbAP5	Nb01	346	37684.12	9.1	37.48	76.65	-0.102	Chloroplast
Nbe02g01650.1	NbAP6	Nb02	480	52339.24	5.7	27.38	88.67	-0.017	Plasma Membrane
Nbe02g08850.1	NbAP7	Nb02	492	52498.39	8.95	33.77	79.25	0	Chloroplast
Nbe02g09470.1	NbAP8	Nb02	438	46972.47	9.35	45.79	79.45	-0.034	Chloroplast
Nbe02g15220.1	NbAP9	Nb02	506	55142.15	6.11	31	90.12	0.048	Chloroplast
Nbe03g02510.1	NbAP10	Nb03	537	58625.69	5.68	38.67	80.56	-0.036	Plasma Membrane
Nbe03g02890.1	NbAP11	Nb03	485	52598.77	6.59	30.75	87.15	-0.064	Plasma Membrane
Nbe03g16130.1	NbAP12	Nb03	533	58197.43	5.82	33.13	80.26	-0.062	Chloroplast
Nbe03g24120.1	NbAP13	Nb03	535	57741.01	5.42	37.99	80.75	-0.096	Plasma Membrane
Nbe03g24140.1	NbAP14	Nb03	232	26049.71	6.22	29.92	81.03	-0.05	Chloroplast
Nbe03g26120.1	NbAP15	Nb03	485	53514.67	5.55	43.06	76.16	-0.101	Vacuole
Nbe03g32770.1	NbAP16	Nb03	512	55484.32	6.22	33.42	81.66	-0.054	Vacuole
Nbe04g05870.1	NbAP17	Nb04	270	29030.16	4.93	22.38	81.59	-0.017	Cytoplasm
Nbe04g11120.1	NbAP18	Nb04	469	50702.21	5.15	42.6	82.75	-0.081	Chloroplast
Nbe04g11140.1	NbAP19	Nb04	539	58217.64	5.26	37.8	81.76	-0.068	Plasma Membrane
Nbe04g11150.1	NbAP20	Nb04	532	59261.09	5.33	35.92	81.48	-0.079	Vacuole
Nbe04g18910.1	NbAP21	Nb04	378	41848.08	9.02	45.36	80.66	-0.222	Chloroplast
Nbe04g21650.1	NbAP22	Nb04	486	52793.87	5.26	35.31	87.2	-0.061	Chloroplast
Nbe04g22250.1	NbAP23	Nb04	548	61207.59	5.37	42.42	75.38	-0.33	Vacuole
Nbe04g22580.1	NbAP24	Nb04	448	48447.8	5.2	40.18	80.49	-0.087	Chloroplast
Nbe04g23860.1	NbAP25	Nb04	483	52529.68	6.06	31.56	87.12	-0.056	Plasma Membrane
Nbe04g24970.1	NbAP26	Nb04	544	59388.5	5.69	39.54	77.17	-0.069	Plasma Membrane
Nbe05g31790.1	NbAP27	Nb05	485	51605.98	5.51	40.3	81.01	0.01	Vacuole
Nbe06g37690.1	NbAP28	Nb06	430	47660.91	9	43.73	82.07	-0.168	Chloroplast
Nbe07g04230.1	NbAP29	Nb07	489	52719.94	5.9	34.16	80.92	0.085	Vacuole
Nbe07g09770.1	NbAP30	Nb07	433	47382.12	8.45	34.81	78.98	-0.203	Endoplasmic Reticulum
Nbe07g12890.1	NbAP31	Nb07	522	56883.53	5.91	45.12	80.13	-0.087	Vacuole
Nbe07g14600.1	NbAP32	Nb07	434	45866.41	8.71	41.19	84.1	0.159	Extracellular
Nbe08g04180.1	NbAP33	Nb08	352	37655.42	4.57	37.87	86.42	0.023	Cytoplasm
Nbe08g07360.1	NbAP34	Nb08	439	47948	9.37	37.43	74.62	-0.112	Chloroplast
Nbe08g14690.1	NbAP35	Nb08	520	56138.67	5.53	42.24	84.71	0.048	Nucleus
Nbe08g16630.1	NbAP36	Nb08	492	53074.03	5.76	31.98	80.63	0.029	Cytoplasm
Nbe08g23030.1	NbAP37	Nb08	501	54059.51	6.24	38.03	79.78	-0.028	Vacuole
Nbe08g26110.1	NbAP38	Nb08	431	47283.94	8.67	37.26	77.08	-0.248	Endoplasmic Reticulum
Nbe08g30480.1	NbAP39	Nb08	523	56923.7	6.12	42.87	81.47	-0.056	Chloroplast
Nbe08g32660.1	NbAP40	Nb08	449	49242.37	9.41	46.4	78.82	-0.106	Chloroplast
Nbe08g33350.1	NbAP41	Nb08	486	52110.22	4.77	37.3	82	-0.074	Chloroplast
Nbe09g12290.1	NbAP42	Nb09	448	48270.55	5.21	46.51	82.72	-0.072	Chloroplast
Nbe09g15450.1	NbAP43	Nb09	362	39899.73	8.62	48.25	82.62	-0.214	Chloroplast
Nbe09g21900.1	NbAP44	Nb09	484	53500.25	8.72	51.9	72.87	-0.167	Chloroplast
Nbe09g25010.1	NbAP45	Nb09	515	54961.46	9.06	31.55	78.99	-0.103	Nucleus
Nbe09g26610.1	NbAP46	Nb09	430	47511.39	5.39	32.1	75.67	-0.299	Nucleus
Nbe09g26620.1	NbAP47	Nb09	360	39373.27	7.96	33.69	70.89	-0.324	Chloroplast
Nbe09g27280.1	NbAP48	Nb09	474	52176.37	6.42	36.58	81.46	-0.101	Chloroplast
Nbe09g29160.1	NbAP49	Nb09	560	61854.23	9.52	36.73	88.59	0.016	Plasma Membrane
Nbe10g07020.1	NbAP50	Nb10	473	52827.44	8.46	34.34	87.78	-0.04	Chloroplast
Nbe10g17450.1	NbAP51	Nb10	458	49889.24	9.16	35.49	78.1	-0.048	Chloroplast
Nbe10g22610.1	NbAP52	Nb10	486	53566.18	7.15	38.93	81.81	-0.124	Vacuole
Nbe11g00900.1	NbAP53	Nb11	481	52407.21	5.55	40.33	82.02	-0.03	Extracellular
Nbe11g16290.1	NbAP54	Nb11	510	55981.21	5.79	36.8	90	-0.005	Vacuole
Nbe11g21770.1	NbAP55	Nb11	385	43056.28	5.31	44.73	90.88	-0.024	Cytoplasm
Nbe11g28230.1	NbAP56	Nb11	497	53266.15	5.25	37.33	86.24	0.053	Extracellular
Nbe12g04330.1	NbAP57	Nb12	488	52927.2	5.82	31.32	88.65	-0.047	Chloroplast
Nbe12g06550.1	NbAP58	Nb12	515	55103.54	9.02	31.26	78.6	-0.121	Nucleus
Nbe12g12170.1	NbAP59	Nb12	499	52972.69	4.87	36.79	88.04	0.102	Plasma Membrane
Nbe12g20610.1	NbAP60	Nb12	511	56170.32	5.92	37.93	88.69	-0.067	Chloroplast
Nbe12g24960.1	NbAP61	Nb12	475	52147.2	6.28	36.53	80.67	-0.124	Chloroplast
Nbe13g10210.1	NbAP62	Nb13	503	53888.5	5.22	34.88	97.28	0.256	Plasma Membrane
Nbe13g15190.1	NbAP63	Nb13	486	53786.39	8.38	48.81	71.98	-0.2	Chloroplast
Nbe13g16280.1	NbAP64	Nb13	485	51723.15	5.82	43.29	80.21	0.007	Vacuole
Nbe14g04460.1	NbAP65	Nb14	503	53677.42	5.53	34.14	97.65	0.268	Plasma Membrane
Nbe14g19390.1	NbAP66	Nb14	552	61139.85	5.8	40.67	77.5	-0.244	Vacuole
Nbe14g23540.1	NbAP67	Nb14	405	45675.34	5.5	42.24	88.32	-0.031	Cytoplasm
Nbe15g06690.1	NbAP68	Nb15	633	69943.84	6.36	48.01	84.87	-0.134	Plasma Membrane
Nbe15g11930.1	NbAP69	Nb15	489	52324.64	4.98	34.74	85.66	-0.055	Chloroplast
Nbe15g12250.1	NbAP70	Nb15	453	49617.49	9.31	47.17	75.76	-0.14	Chloroplast
Nbe16g03730.1	NbAP71	Nb16	557	61725.7	6.18	37.73	76.79	-0.237	Vacuole
Nbe16g06900.1	NbAP72	Nb16	508	54766.04	5.34	32.68	81.93	-0.032	Chloroplast
Nbe16g11100.1	NbAP73	Nb16	434	46057.62	8.48	42.77	83	0.135	Extracellular
Nbe16g12920.1	NbAP74	Nb16	417	45411.24	8.64	37.62	85.76	-0.084	Cytoplasm
Nbe16g16160.1	NbAP75	Nb16	666	74023.16	5.73	50.48	82.25	-0.186	Plasma Membrane
Nbe16g29310.1	NbAP76	Nb16	358	39716.66	8.7	41.46	80.56	-0.227	Nucleus
Nbe17g12360.1	NbAP77	Nb17	473	52924.74	8.71	36.23	89.41	-0.026	Cytoplasm
Nbe17g16820.1	NbAP78	Nb17	451	49437.74	8.46	32.88	71.15	-0.289	Nucleus
Nbe17g21730.1	NbAP79	Nb17	333	35011.68	6.78	49.24	80.24	0.087	Chloroplast
Nbe17g23660.1	NbAP80	Nb17	423	45957.4	5.19	37.59	97.21	0.222	Vacuole
Nbe17g23670.1	NbAP81	Nb17	423	45881.31	5.19	37.59	97.21	0.223	Vacuole
Nbe17g23680.1	NbAP82	Nb17	421	45697.07	5.19	37.72	96.51	0.21	Vacuole
Nbe17g23690.1	NbAP83	Nb17	423	45881.31	5.19	37.59	97.21	0.223	Vacuole
Nbe17g23700.1	NbAP84	Nb17	421	45723.15	5.19	37.72	97.43	0.221	Vacuole
Nbe17g23710.1	NbAP85	Nb17	423	45941.4	5.19	37.14	97.21	0.231	Vacuole
Nbe17g23720.1	NbAP86	Nb17	423	45881.31	5.19	37.59	97.21	0.223	Vacuole
Nbe19g12400.1	NbAP87	Nb19	648	71668.45	5.42	50.71	87.53	-0.197	Plasma Membrane
Nbe19g34610.1	NbAP88	Nb19	472	50707.7	4.76	49.07	79.32	-0.035	Chloroplast
Nbe19g34630.1	NbAP89	Nb19	567	62957.51	5.55	43.92	84.94	-0.236	Nucleus

### 3.2 Phylogenetic analysis

**Figure 1:**
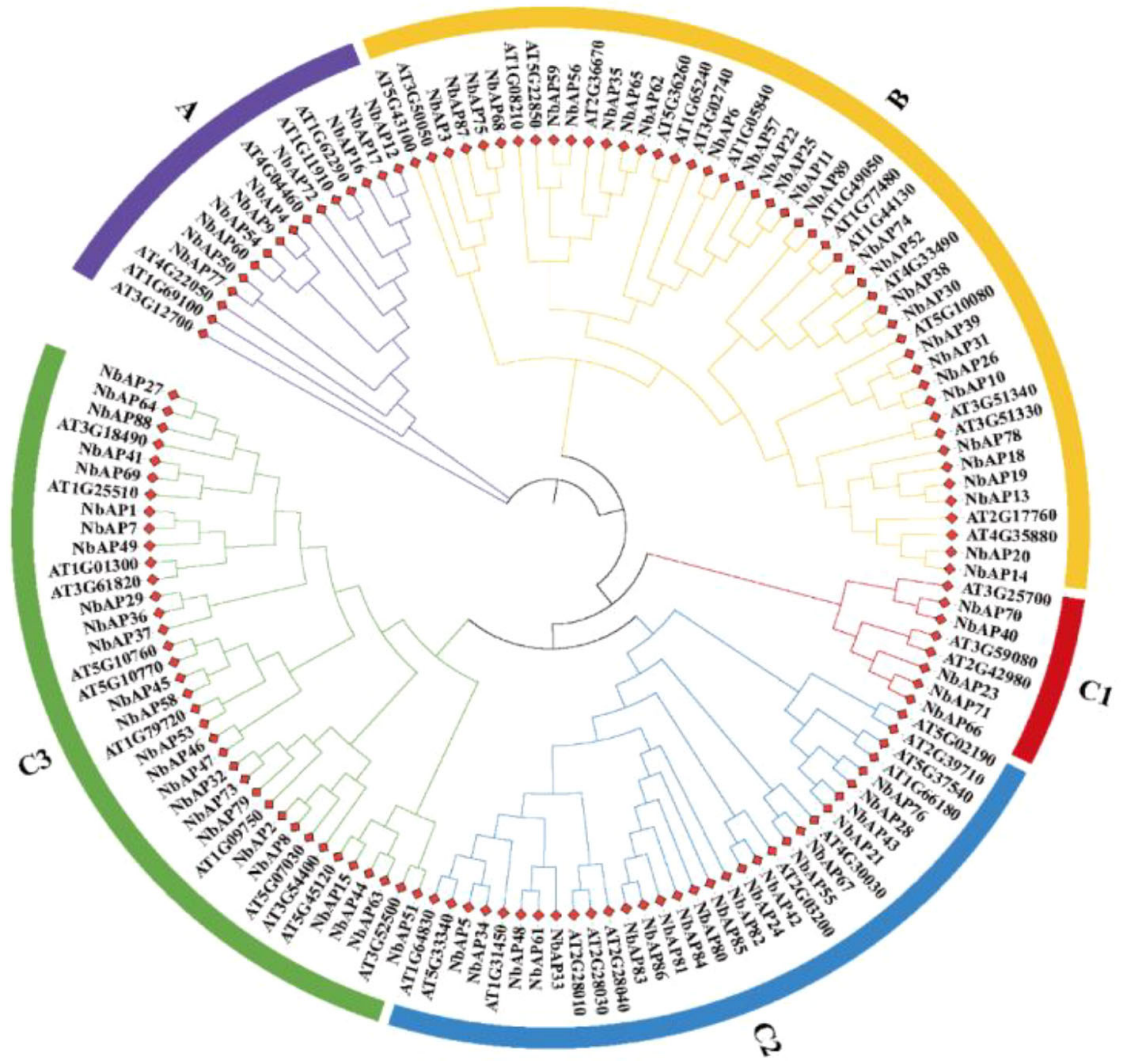
Phylogenetic analysis of the APs in *Nicotiana benthamiana* and *Arabidopsis thaliana*. Group A means typical APs, Group B means Nucellin-like APs, Group C1-C3 means Atypical APs.

To elucidate the evolutionary relationships of *N. benthamiana* NbAPs, this study constructed a phylogenetic tree incorporating homologous genes from *Arabidopsis thaliana* (**Figure 1**). The NbAP gene family in *N. benthamiana* can be clearly divided into three major clades: Typical APs (Group A) contain 10 members, all possessing the conserved Plant-Specific Insert (PSI) domain, and homologous genes from both species (e.g., *NbAP72*/AT4G04460) cluster together, indicating functional conservation. Nucellin-like APs (Group B) consist of 39 members (e.g., *NbAP89*), characterized by carrying QCDYE and GCGYDQ cysteine sequence motifs, and form an independent branch with *Arabidopsis* genes (e.g., AT1G49050). With reference to existing research (Wang et al., 2024), Atypical APs (Group C) is the largest clade (40 members), further subdivided into three subgroups based on structural differences, with C1 having the fewest members (5 members), followed by C2 (14 members), and C3 having the most members (21 members). Within the C3 subgroup, genes *NbAP27, NbAP64, NbAP88* cluster closely with *Arabidopsis* AT3G18490 (*ASPG1*), suggesting they may share role in seed dormancy, viability, and germination. In the C2 subgroup, *NbAP24*, and *NbAP42* genes cluster closely with *Arabidopsis* AT2G03200 (*ASPR1*), implying their important function in root development. Additionally, we observed that *NbAP29, NbAP36, NbAP37* in the C3 subgroup did not cluster closely with any corresponding *Arabidopsis* genes, suggesting these three genes may have novel functions. Overall, the phylogenetic tree reveals the evolutionary conservation of APs genes between species, while the differentiation of APs among different species may correspond to diverse biological functions.

### 3.3 Conserved motifs, protein, and gene structure analysis

**Figure 2:**
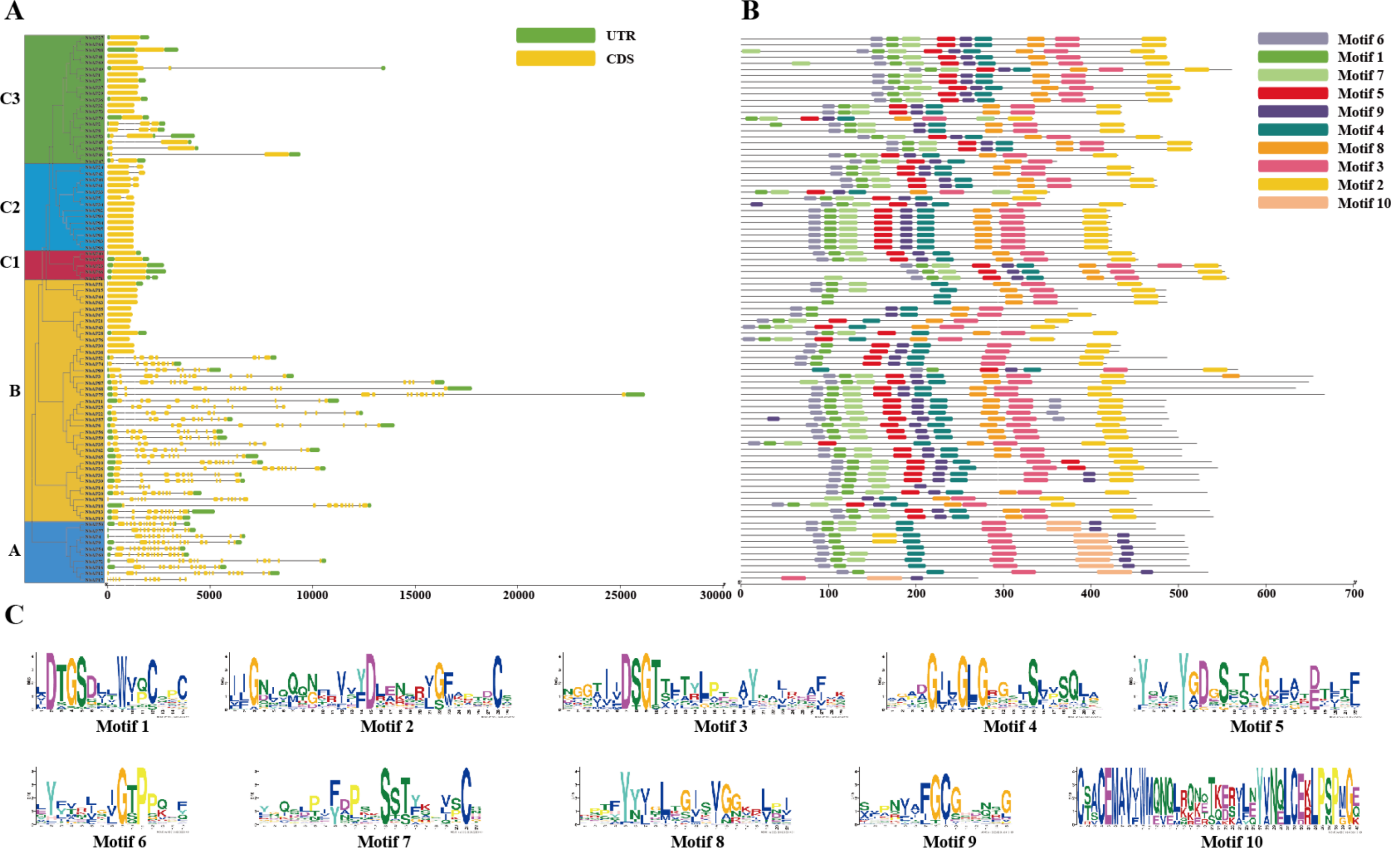
Gene structure and conserved protein motif analysis of the NbAP family in *N. benthamiana.* **(A)** Exon-intron structure of 89 *NbAP* genes. Yellow boxes represent exons, and black lines represent introns. The lengths of genes and structural components are scaled accordingly. **(B)** Distribution of ten conserved motifs (Motif 1-10) in NbAP proteins, identified by MEME suite. Each motif is represented by a colored box, as indicated in the legend. **(C)** Sequence logos of the ten identified motifs, showing the conservation of amino acids at each position.

Analysis of the structure of the 89 *NbAP* genes (**Figure 2A**) revealed significant diversity in their exon numbers, ranging from 1 to 14. The number of exons in the Atypical AP subfamily was highly conserved, ranging from 1 to 3, with all members of the C1 subfamily containing only a single exon and lacking introns. In contrast, typical AP subfamily genes generally contained large number of exons (11–14), e.g., *NbAP12* contained 14 exons, *NbAP17* contained 11 exons. Genes within the same subfamily had similar exon numbers, suggesting relatively conserved functions within subfamilies. Interestingly, the Nucellin-like protease subfamily exhibited variation in exon number (1–13), e.g., *NbAP51* contained 1 exon, while *NbAP75* contained 13 exons, hinting at potential functional diversity within this subfamily’s members.

Ten conserved motifs (Motif 1-10) were identified in NbAP proteins using MEME software (**Figures 2B, C**). We found that Motif 1, 3, 4, 6, and 9 were ubiquitous across the entire NbAP family, indicating these motifs play a role in the conserved functions of the NbAP family. Motif 2 was less distributed (20%) in the Typical NbAP subfamily, found only in the gene sequences of *NbAP4* and *NbAP9*. Interestingly, we did not find Motif 5 and Motif 8 in the Typical NbAPs subfamily, while these two motifs were present in other subfamilies. Conversely, members of the Typical NbAP subfamily specifically contained Motif 10, which was absent in all other subfamilies. It can be speculated that Motif 10 plays an important role in the functional differentiation of the Typical AP family genes.

### 3.4 Gene duplication and chromosomal distribution analysis

**Figure 3:**
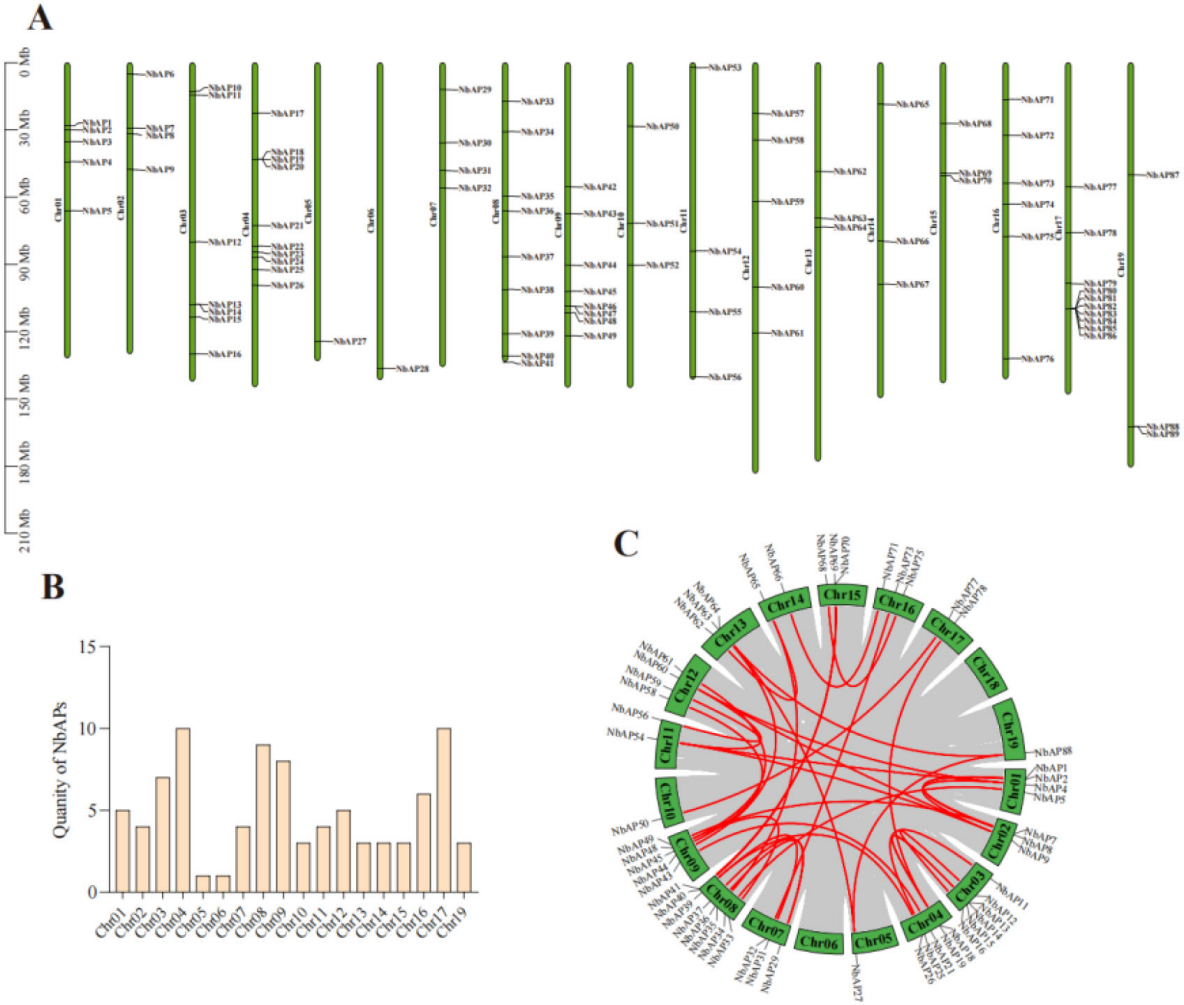
Chromosomal distribution and duplication events of *NbAP* genes in *N. benthamiana*. **(A)** Physical locations of the 89 *NbAP* genes on the 19 chromosomes. The chromosome number is indicated at the top of each bar. The scale on the left represents chromosome length (Mb). Gene names are listed on the right side of their corresponding chromosomal positions. Tandemly duplicated gene clusters are highlighted in red. **(B)** Bar chart showing the number of *NbAP* genes located on each chromosome. **(C)** Inter-chromosomal relationships of segmentally duplicated *NbAP* genes. The chromosomes are represented by gray bars. The red curves connect the segmentally duplicated gene pairs, with the corresponding gene names labeled nearby.

Members of the NbAP gene family are unevenly distributed across 19 chromosomes of *N. benthamiana* (**Figures 3A, B**). The chromosomal distribution density varied significantly: with Chr4 and Chr17 each contain 10 genes, Chr8 contain 9 genes, Chr9 contain 8 genes, Chr3 contain 7 genes, Chr16 contain 6 genes, Chr1 and Chr12 each contain 5 genes, while Chr2, Chr7, and Chr11 each contain 4 genes. Additionally, Chr10, Chr13, Chr14, Chr15, and Chr19 each contain 3 genes, whereas Chr5, and Chr6 possess only a single gene. Furthermore, we found that some NbAP members exist in gene clusters on chromosomes, such as *NbAP80–86* clustered on chromosome 17, which may suggest these genes cooperatively participate in the same biological process.

Segmental duplication is the main driving force for the expansion of the NbAP family, promoting the increase of functionally similar genes within the same subfamily (e.g., redundant backup of disease resistance-related genes). We identified 36 segmental duplication events involving 56 genes (approximately 62.9% of the total family members) (**Figure 3C**). The Ka/Ks ratio is a crucial indicator in molecular evolutionary studies used to infer the type of natural selection acting on protein-coding genes. We found that all duplication events occurred within NbAP subfamilies, indicating specific gene expansion within subfamilies. However, significant differences existed between subfamilies: the Typical AP subfamily contained 8 duplication events, the Atypical AP subfamily contained 18, and the Nucellin-like AP subfamily contained 10. The Ka/Ks value for the *NbAP13/NbAP18* gene pair was 0.75, suggesting that some sites may have undergone adaptive evolution, potentially accompanied by subfunctionalization or neofunctionalization (**Table 2**).

**Table 2:** Ratios of nonsynonymous (Ka) and synonymous (Ks) of NbAPs gene fragment duplication pairs in Nicotiana benthamiana.

Gene pairs	Ka	Ks	Ka/Ks
NbAP1/NbAP7	0.01435	0.12535	0.11448
NbAP2/NbAP8	0.010194	0.130182	0.078305
NbAP4/NbAP9	0.024564	0.063868	0.384597
NbAP4/NbAP54	0.113642	0.460581	0.246736
NbAP4/NbAP60	0.116821	0.52858	0.22101
NbAP5/NbAP34	0.031299	0.138589	0.225839
NbAP7/NbAP49	0.149776	1.194502	0.125388
NbAP9/NbAP54	0.10572	0.481586	0.219524
NbAP9/NbAP60	0.108855	0.569385	0.191179
NbAP11/NbAP25	0.015063	0.059196	0.254463
NbAP12/NbAP16	0.070916	0.551697	0.128542
NbAP13/NbAP18	0.066461	0.088199	0.753537
NbAP14/NbAP19	0.447695	2.722935	0.164416
NbAP18/NbAP78	0.302321	0.707829	0.42711
NbAP21/NbAP43	0.016401	0.14516	0.112984
NbAP26/NbAP39	0.361602	1.992568	0.181475
NbAP27/NbAP64	0.020548	0.159159	0.129102
NbAP27/NbAP88	0.173662	0.913252	0.190158
NbAP29/NbAP36	0.029979	0.232172	0.129122
NbAP29/NbAP37	0.158711	1.169162	0.135748
NbAP31/NbAP39	0.020739	0.060782	0.341205
NbAP32/NbAP73	0.010317	0.152508	0.067652
NbAP33/NbAP48	0.230652	0.858621	0.26863
NbAP35/NbAP65	0.12195	0.39266	0.310574
NbAP40/NbAP70	0.021584	0.197777	0.109134
NbAP41/NbAP69	0.020964	0.147503	0.142126
NbAP44/NbAP63	0.023121	0.129153	0.179024
NbAP45/NbAP58	0.022022	0.121492	0.181266
NbAP48/NbAP61	0.029066	0.102286	0.284166
NbAP50/NbAP77	0.032872	0.083041	0.395847
NbAP54/NbAP60	0.02448	0.103453	0.236627
NbAP56/NbAP59	0.016236	0.119788	0.135543
NbAP62/NbAP65	0.026133	0.080521	0.324545
NbAP64/NbAP88	0.174484	0.976612	0.178662
NbAP66/NbAP71	0.019022	0.168226	0.113076
NbAP68/NbAP75	0.021423	0.070247	0.30496

### 3.5 Collinearity analysis

**Figure 4:**
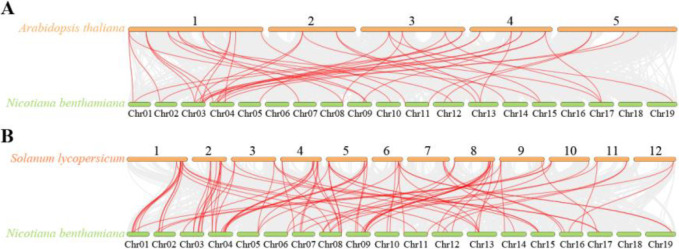
Comparative synteny analysis of aspartic protease genes between *N. benthamiana* and two reference species. **(A)** Syntenic relationships between *N. benthamiana* and *A. thaliana*. Gray lines in the background indicate the overall collinear blocks between the two genomes, while red lines highlight the collinear pairs specifically involving *NbAP* genes. **(B)** Syntenic relationships between *N. benthamiana* and *S. lycopersicum*. Similarly, gray lines show the background genomic collinearity, and red lines emphasize the collinear *NbAP* gene pairs.

Collinearity analysis of the *N. benthamiana* NbAP gene family with other species revealed significant differences in evolutionary relationships. In the comparison with tomato (*Solanum lycopersicum*), 63 gene members showed collinearity, forming 85 collinear gene pairs, with relatively high conservation of chromosomal positions (**Figure 4B**). This large-scale conservation suggests stable genomic structure maintenance after the divergence of the two species, likely due to their relatively close phylogenetic relationship (both belonging to Solanaceae), and implies these genes constitute core functional modules involved in important biological processes. In contrast, collinearity between *N. benthamiana* and *Arabidopsis thaliana* was significantly weaker, with only 30 genes forming 43 collinear gene pairs, and chromosomal position conservation was very low (**Figure 4A**). This result reflects the extensive genome rearrangements experienced by Solanaceae and Brassicaceae plants during their long independent evolution, leading to the fragmentation of collinear segments.

### 3.6 Promoter cis-element analysis

**Figure 5:**
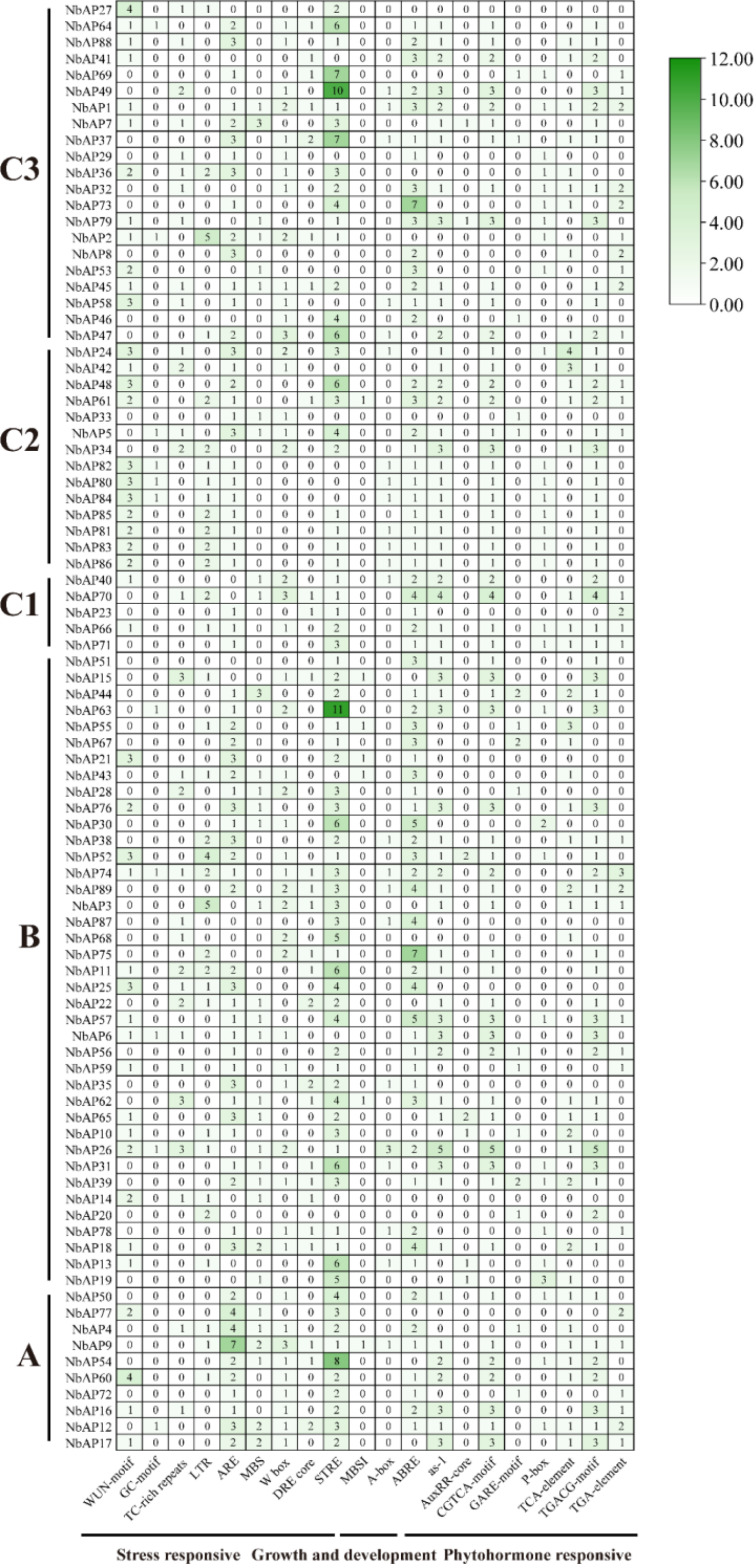
Analysis of cis-acting elements in the promoters of *NbAP* genes. The predicted cis-acting elements are categorized into three major functional classes: Phytohormone Response, Stress Response, and Growth and Development. Each colored box represents an individual element, and its position in the horizontal bar indicates the relative location in the promoter region. Key elements such as the wound-responsive WUN-motif and the stress-responsive STRE are prominently distributed among the family members, suggesting their potential roles in environmental adaptation and defense responses.

With reference to existing research (Pan et al., 2024), a total of 20 types of cis-acting elements were identified in the promoter regions and categorized into three major functional classes (**Figure 5**): Stress Response Elements (including WUN-motif, GC-motif, TC-rich repeats, LTR, ARE, MBS, W box, DRE core, STRE), Growth and Development related elements (MBSI, A-box, ABRE), and Phytohormone Response Elements (as-1, AuxRR-core, CGTCA-motif, GARE-motif, P-box, TCA-element, TGACG-motif, TGA-element). The presence of diverse cis-elements in NbAP family members indicates their potential involvement in varied biological functions related to plant development and environmental responses. The WUN-motif is an important wound-inducible cis-acting element in plant gene promoters, specifically activating the expression of downstream defense-related genes upon mechanical damage or pathogen infection. Therefore, we speculate that NbAP members containing this element might play a role during *Agrobacterium* transient infection of *N. benthamiana*. Analysis results showed that *NbAP27, NbAP58, NbAP24, NbAP48, NbAP82, NbAP80, NbAP84, NbAP21, NbAP52, NbAP25, NbAP60* each contained no less than 3 WUN-motifs, suggesting these genes might be important players in the *Agrobacterium* infection process. Another element found in high numbers was STRE; for instance, the *NbAP63* promoter contained 11, *NbAP49* contained 10, and *NbAP54* contained 8, hinting at the potential biological functions of these genes in stress response.

### 3.7 Expression patterns of NbAP family members after Agrobacterium infection of N. benthamiana

**Figure 6:**
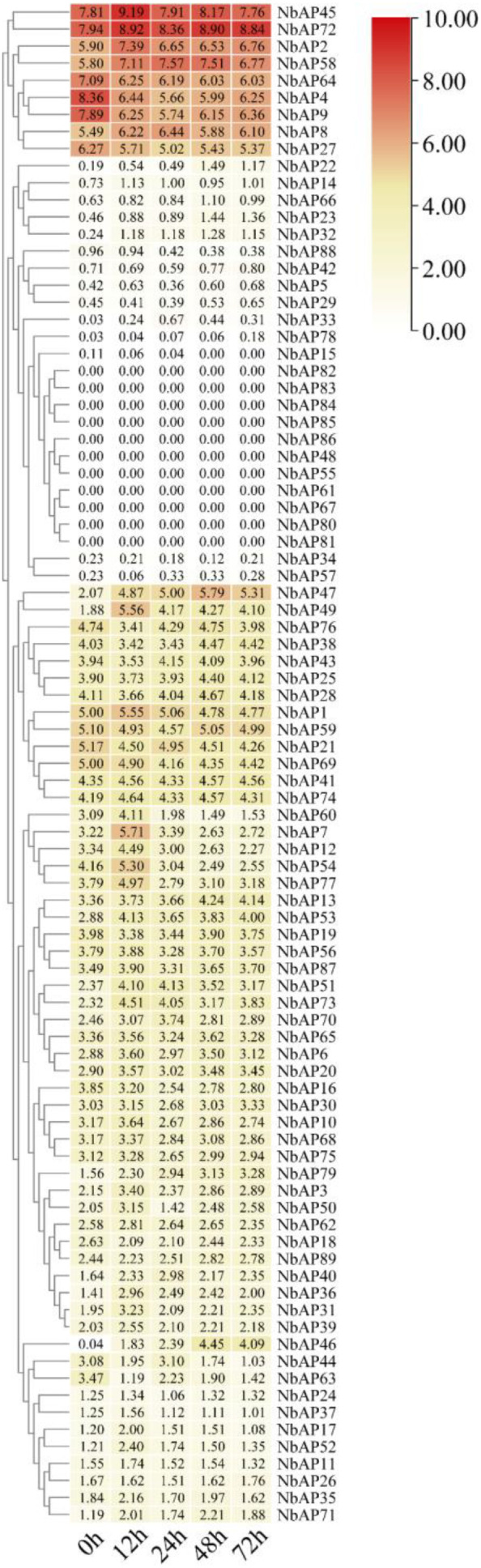
Expression profiles of *NbAP* genes in response to *Agrobacterium* infiltration. Expression patterns of *NbAP* genes at different time points (0, 12, 24, 48, 72 hours post-infiltration. The heatmap displays log2-transformed FPKM values, with colors ranging from yellow (low expression) to red (high expression). Genes are clustered based on their expression trends, revealing distinct temporal response patterns.

To elucidate the role of the NbAP family in the *N. benthamiana* bioreactor, this study analyzed the dynamic expression of NbAP family members at different time points (0h, 12h, 24h, 48h, 72h) post-*Agrobacterium* infiltration using public transcriptome data(NCBI under accession no. ERP140419) (Wang et al., 2024)(**Figure 6**). The main findings are as follows: Differential temporal response characteristics were observed. Early high-response genes included *NbAP7, NbAP36, NbAP53, NbAP49*, and *NbAP22*, which were rapidly activated at 12 hours post-infiltration (hpi) and maintained relatively high expression, suggesting their involvement in early antiviral signaling initiation. Sustained activation genes included *NbAP46, NbAP47*, and *NbAP79*, whose expression continuously increased from 0 to 72 hpi, reflecting their important functions during the infection process. Consistently low-expression genes included *NbAP60*. To further validate these results, we performed RT-qPCR on leaves harvested 12 hours after *Agrobacterium* infiltration. The results confirmed that *NbAP46, NbAP47*, and *NbAP79* significantly responded to *Agrobacterium* infection (**Supplementary Figure 1**). The changes in transcript levels of these genes provide candidate genes for experimentally verifying their role in recombinant protein degradation.

### 3.8 Validation of *NbAP* family members’ involvement in recombinant protein stability via VIGS

**Figure 7:**
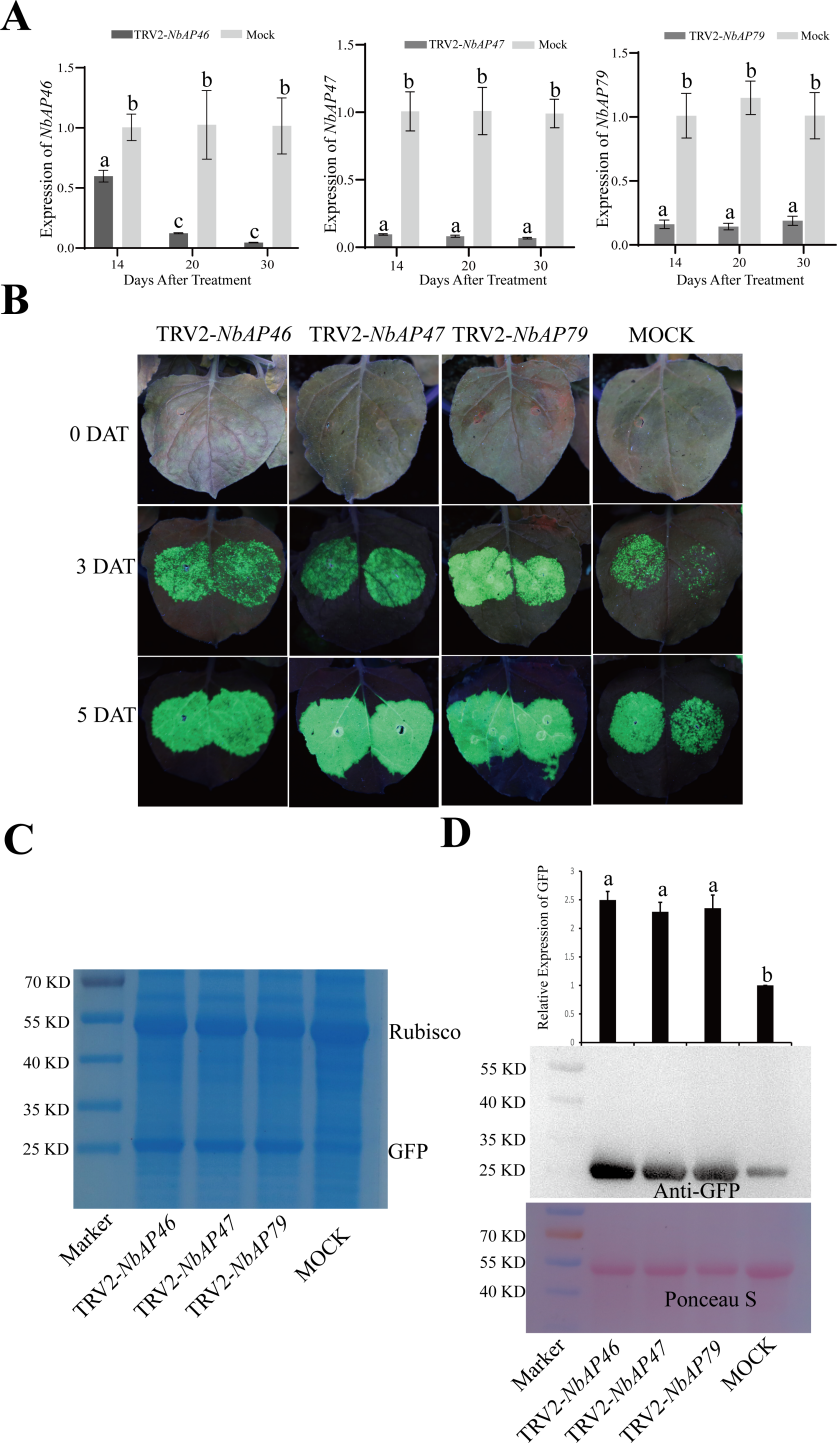
Functional validation of selected *NbAPs* in recombinant protein accumulation via virus-induced gene silencing. **(A)** Relative expression levels of *NbAP46*, *NbAP47*, and *NbAP79* in Mock (TRV2:TRV1) and VIGS-silenced plants at 14, 20, and 30 days post-agroinfiltration (dpai), as determined by RT-qPCR. Different letters above columns indicate significant`` differences based on Duncan’s multiple range test (P < 0.05). Data are shown as the mean ± SD of three independent experiments. **(B)** *In vivo* monitoring of GFP fluorescence in control and VIGS-silenced plants at 3 and 5 days post-infiltration (dpi) with Agrobacterium carrying the *GFP* gene. **(C)** Coomassie Brilliant Blue staining analysis of GFP protein accumulation in leaf tissues harvested at 3 dpi. **(D)** Western blot analysis of GFP protein accumulation in leaf tissues harvested at 3 dpi. Different letters above columns indicate significant differences based on Duncan’s multiple range test (P < 0.05). Densitometry of western blots Data are shown as the mean ± SD of three independent experiments.

To investigate the biological roles of *NbAP* genes in transient protein expression in *N. benthamiana*, we selected three genes (*NbAP46, NbAP47, NbAP79*) that exhibited sustained and pronounced activation post-*Agrobacterium* infection for functional validation. First, these three genes were individually silenced using VIGS technology. RT-qPCR analysis at 14, 20, and 30 days post-treatment showed inhibited expression of all three genes (**Figure 7A**). The positive plant materials obtained via VIGS were named TRV2-*NbAP46*, TRV2-*NbAP47*, and TRV2-*NbAP79*, respectively. To assess the effect of NbAPs silencing on transient protein accumulation, the GFP reporter gene was infiltrated into both VIGS-silenced and control (TRV2:TRV1) plants. GFP fluorescence was monitored at 3 and 5 days post-infiltration. The results showed that, compared with the control, GFP protein expression was significantly enhanced in TRV2-*NbAP46*, TRV2-*NbAP47*, and TRV2-*NbAP79* plants (**Figure 7B**). We further validated these results by performing coomassie brilliant blue staining (**Figure 7C**) and Western blot (**Figure 7D**) analysis on plant materials harvested 3 days post-infiltration. Consistent with the observed phenotype, TRV2-*NbAP46*, TRV2-*NbAP47*, and TRV2-*NbAP79* plants had more GFP protein expression compared to the control. This indicates that *NbAP46*, *NbAP47*, and *NbAP79* likely predicted aspartic proteases in the degradation of recombinant proteins during transient expression.

## 4 Discussion

In this study, we performed a systematic genome-wide identification, evolutionary analysis, and functional characterization of the aspartic protease (AP) gene family in *N. benthamiana*. Our findings reveal its putative roles in plant growth, development, stress responses, and notably, as a key regulatory factor influencing recombinant protein stability in plant molecular farming.

A total of 89 NbAP members were identified in *N. benthamiana*, which exhibited considerable diversity in both physicochemical properties and subcellular localization. Notably, the majority of NbAPs were localized to the vacuole (28%) and chloroplasts (35%), consistent with their fundamental roles in protein degradation, autophagy, and stress responses. Furthermore, several NbAP members, including *NbAP32* and *NbAP53*, were predicted to localize extracellularly, suggesting their potential to directly degrade recombinant proteins in the secretory pathway, thereby representing a significant risk factor for protein loss in plant bioreactor systems. Phylogenetic analysis classified NbAPs into three major categories: typical, nucellin-like, and atypical APs, with the latter further subdivided into three subgroups. Comparative clustering with *A. thaliana* homologs revealed that certain NbAP members, such as *NbAP27* and *NbAP64*, are homologous to *AtASPG1* and may be involved in seed germination and dormancy regulation, while *NbAP24* and *NbAP42*, homologous to *AtASPR1*, potentially function in root development. Interestingly, several members, including *NbAP29* and *NbAP36*, did not cluster closely with any *Arabidopsis* genes, suggesting potential functional divergence from characterized Arabidopsis APs in *N. benthamiana*.

Analyses of gene structure and conserved motifs provided further evidence for both functional conservation and divergence among AP subfamilies. Typical APs contained a higher number of exons (11–14) and possessed a unique Motif 10, potentially associated with their characteristic plant-specific insert (PSI) domain and vacuolar sorting functions. In contrast, atypical APs generally contained fewer exons (1–3) but retained core catalytic motifs (e.g., Motif1, 3, 4, 6, and 9), indicating the preservation of essential proteolytic functions during evolution. Gene duplication and collinearity analyses revealed that segmental duplication serves as the primary driving force for NbAP family expansion, with all duplication events occurring within subfamilies, further reinforcing functional redundancy and subfunctionalization. High collinearity with tomato (*Solanum lycopersicum*) reflects genomic conservation within Solanaceae species, whereas low collinearity with *A. thaliana* suggests substantial genomic rearrangements during their evolutionary divergence. Promoter cis-element analysis indicated that NbAP members are broadly involved in stress responses, hormone signaling, and growth and developmental processes. Multiple members contained stress-responsive elements such as WUN-motif and STRE. Particularly in the context of *Agrobacterium* infiltration, genes such as *NbAP27* and *NbAP58* may function in early defense responses. This prediction was strongly supported by expression profiling: following *Agrobacterium* infection, genes such as *NbAP36* and *NbAP7* exhibited rapid early induction, whereas *NbAP46, NbAP47*, and *NbAP79* showed sustained upregulation, suggesting their persistent involvement during the infection process. Functional validation using Virus-Induced Gene Silencing (VIGS) targeting the highly expressed *NbAP46, NbAP47*, and *NbAP79* demonstrated that silencing these genes significantly enhanced the accumulation of GFP reporter protein. Western blot analysis further confirmed these observations, indicating that these three genes likely negatively regulate the stability of foreign proteins through direct or indirect proteolytic mechanisms. This finding not only provides new insights into the mechanisms of recombinant protein degradation in plants but also identifies potential targets for optimizing the *N. benthamiana* bioreactor platform through gene editing or silencing strategies. In subsequent studies, we will investigate the enzymatic properties and substrate specificity of the target proteins, with a focus on elucidating potential differences in the specificity of NbAPs in suppressing protein degradation. Furthermore, we’ll employ CRISPR gene editing technology to knock out the identified target genes, aiming to develop a universal chassis applicable to PMF. These studies hold broad application prospects in the field of recombinant protein expression.

Research on *Nicotiana tabacum* (Wang et al., 2024) and our findings jointly revealing the conservation and species specificity of the AP family in Solanaceae plants. Functionally, however, Wang et al.’s study focused on the critical roles of AP family members in stem vascular tissue development, xylem PCD, and lignin synthesis, whereas we addressed the bottleneck in *N. benthamiana* as a molecular farming platform by highlighting the sustained high expression of specific NbAPs after Agrobacterium infiltration and validating their negative regulatory effects on the stability of foreign recombinant proteins through VIGS silencing. This underscores how the same gene family may play distinctly different physiological roles in different species or research systems. Additionally, consistent with previous studies, plant APs are known to play critical roles in various plant physiological processes, including xylem development, programmed cell death (PCD), and stress adaptation. For instance, *PtAP66* and *PtAP17* could function in wood formation in poplar (Cao et al., 2019). These functions suggest that, in addition to directly degrading foreign proteins, NbAPs may also indirectly influence recombinant protein accumulation by modulating host cellular states.

In conclusion, this study provides the first comprehensive characterization of the diversity, evolutionary features, and expression regulatory patterns of the AP gene family in *N. benthamiana*, and preliminarily validates the role of selected members in regulating recombinant protein stability. Future research should focus on elucidating the enzymatic properties, substrate specificity, and precise mechanisms of key NbAPs in protein degradation pathways, thereby facilitating the development of more precise strategies for enhancing recombinant protein production in plant bioreactors.

## Data Availability

The original contributions presented in the study are included in the article/**Supplementary Material**. Further inquiries can be directed to the corresponding authors.

## Supplementary material

Supplementary Figure 1 The expression of *NbAP46*, *NbAP47*and *NbAP79* was verified by RT-qPCR
