# Supplementary figures and images for: Analysis of the aspartic protease gene family in Nicotiana benthamiana and its application in recombinant protein expression
Source: Front Plant Sci. 2026 Mar 16;17:1778448. doi: 10.3389/fpls.2026.1778448 (PMC13033640; doi:10.3389/fpls.2026.1778448)

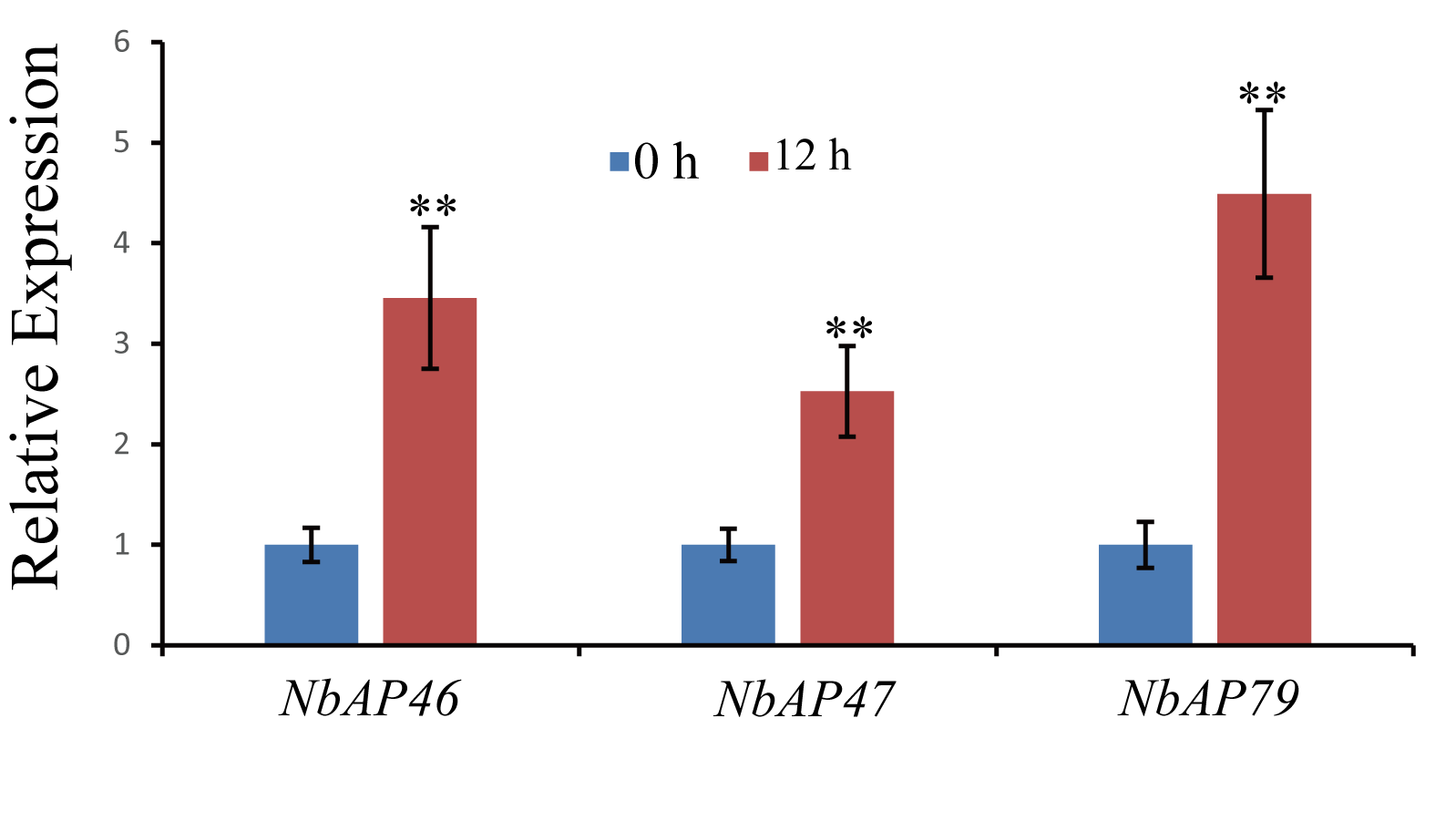

Supplement: Supplementary Figure 1 — The expression of NbAP46, NbAP47and NbAP79 was verified by RT-qPCR [file Image1.tif]
